# Personally addressed hand-signed letters increase questionnaire response: a meta-analysis of randomised controlled trials

**DOI:** 10.1186/1472-6963-6-111

**Published:** 2006-09-05

**Authors:** Pippa Scott, Phil Edwards

**Affiliations:** 1 Department of Epidemiology and Population Health, London School of Hygiene & Tropical Medicine, Keppel Street, London, UK

## Abstract

**Background:**

Postal questionnaires are commonly used to collect data for health studies, but non-response reduces study sample sizes and can introduce bias. Finding ways to increase the proportion of questionnaires returned would improve research quality. We sought to quantify the effect on response when researchers address participants personally by name on letters that accompany questionnaires.

**Methods:**

All randomised controlled trials in a published systematic review that evaluated the effect on response of including participants' names on letters that accompany questionnaires were included. Odds ratios for response were pooled in a random effects meta-analysis and evidence for changes in effects over time was assessed using random effects meta-regression.

**Results:**

Fourteen randomised controlled trials were included covering a wide range of topics. Most topics were unrelated to health or social care. The odds of response when including participants' names on letters were increased by one-fifth (pooled OR 1.18, 95% CI 1.03 to 1.34; p = 0.015). When participants' names and hand-written signatures were used in combination, the effect was a more substantial increase in response (OR 1.45, 95% CI 1.27 to 1.66; p < 0.001), corresponding to an absolute increase in the proportion of questionnaires returned of between 4% and 10%, depending on the baseline response rate. There was no evidence that the magnitude of these effects had declined over time.

**Conclusion:**

This meta-analysis of the best available evidence indicates that researchers using postal questionnaires can increase response by addressing participants by name on cover letters. The effect appears to be enhanced by including hand-written signatures.

## Background

Mailed questionnaires are often used in health research to collect data. However, non-response reduces study sample sizes and can introduce bias [1]. Strategies that appear to increase response have been identified by a systematic review and include making questionnaires and accompanying letters more personal. In the published systematic review, all studies of personalisation were combined in one analysis [2]. The question of whether different methods of personalisation (such as hand-written signatures or including a participant's name on the cover letter) produce different effects on response was not examined. Recently, a randomised controlled trial and meta-analysis of previous trials found no advantage in hand-signed letters compared to those with photocopied or scanned signatures [3]. The study did not investigate the effect of using the participant's name, or using a hand-written signature and the participant's name together.

## Methods

All randomised controlled trials that evaluated the inclusion of participants' names on the letters accompanying postal questionnaires were identified from a published systematic review [2]. There was no restriction by language, questionnaire topic, or study population. The search criteria used are described in detail in the published report which was last updated in 2003 [2]. Only studies in which all participants were known to be randomly allocated to intervention groups were included. We extracted data from each study on the year of publication, numbers of participants randomised and numbers responding. We pooled the odds ratios for response estimated by each trial in a random effects meta-analysis [4] and tested for heterogeneity in effects using the chi-squared statistic [4] and the *I*^2 ^statistic [5]. Evidence for systematic differences between small and large trials (e.g. publication bias) was assessed using Egger's test [6]. We hypothesised *a priori *that due to increased use of these methods through electronic means, the size of the effects of personalisation on response have decreased over time as people become desensitised to the methods. We examined the evidence for this hypothesis by conducting a random-effects meta-regression of the estimated effects on year of publication. A residual maximum likelihood was used to estimate the between-study component of variance.

## Results

Fourteen randomised controlled trials including 12,102 participants were identified from the published systematic review (additional file 1: table 1) [7-20]. Further details about the numbers of records of potentially eligible studies retrieved by the search strategy are reported elsewhere [2]. Study participants were individuals from professional groups and members of the public, and included non-respondents to previous mailings of questionnaires. Thirteen trials were conducted in the US and one was conducted in New Zealand. None of the included trials reported using recorded delivery.

**Figure 1:**
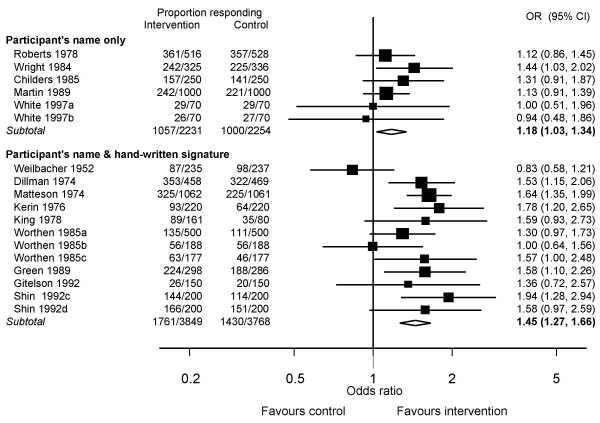
Odds ratios for questionnaire response associated with the inclusion of participants' names on covering letters.

We found evidence for a small but statistically significant increase in response when participants were personally addressed by name and signatures were not written by hand on covering letters (pooled OR 1.18, 95% CI 1.03 to 1.34; p = 0.015; figure 1). There was no evidence for changes in this effect over time (regression coefficient = -0.006, 95% CI -0.04 to 0.028; p = 0.64). We found strong evidence for an increase in response when participants were personally addressed by name and signatures were hand-written, compared with when neither method of personalisation was used (pooled OR 1.45, 95% CI 1.27 to 1.66; p < 0.001). There was little evidence for a change in this effect on response over time (regression coefficient = 0.011, 95% CI -0.001 to 0.024; p = 0.068). Heterogeneity between trial results (unconditional on year of publication) was not statistically significant in either analysis (p = 0.753, *I*^2 ^= 0.0% and p = 0.108, *I*^2 ^= 35% respectively), even when the higher threshold for significance (p < 0.10) was used.[5] There was no evidence for systematic differences between small and large trials (p = 0.845 and p = 0.600 respectively). Heterogeneity conditional on year of publication remained non-significant in both analyses.

## Discussion

Including participants' names and hand-written signatures on letters sent with postal questionnaires appears to increase response, compared with using neither method. The use of participants' names in the absence of a hand-written signature may also improve response, but the effect appears to be smaller. The magnitude of these effects was hypothesised to have declined over recent years but we found no evidence that this was the case.

### Strengths and weaknesses of the study

Although the types of participants and questionnaire topics varied between the included studies, the majority showed an increase in response with these methods of personalisation. We found no evidence for systematic differences between small and large studies. The trials for our study were those previously identified by a systematic review. However, this review was up-to-date and had used explicit search criteria.[2] The majority of the included studies were conducted in the US and none was conducted in a lower income country. Furthermore, most questionnaire topics were unrelated to health or social care. Both of these points may reduce the generalisability of these findings to other settings, but this remains a matter for judgement. None of the included studies reported the method of allocation to intervention groups, although all stated that randomisation was used. We were therefore unable to investigate whether the exclusion of trials with inadequate allocation concealment would have significantly altered our results. One included trial found a marked reduction in response with personalisation. This trial had asked alumni members about their home and business addresses, and business positions held. This might suggest that a personal address on cover letters could be detrimental to response, should the participants perceive that their peers will have access to their personal information. We did not investigate any effects that personalisation may have had on the accuracy and validity of the data collected. We are therefore unable to say whether such personalisation affects the quality of response, only that it appears to increase the quantity of response.

### Implications for researchers and further research

Our results suggest that researchers who go to the trouble of hand-signing letters can increase response if they also personally address participants by name. An absolute increase in the proportion of questionnaires returned of between 4% and 10% can be expected, depending on the baseline response proportion when using neither intervention [2]. Currently there is insufficient evidence to say whether high quality scanned signatures have similar effects to hand-written signatures when used on letters in conjunction with participants' names. Randomised controlled trials comparing scanned with hand-written signatures are needed.

## Conclusion

This meta-analysis of the best available evidence indicates that researchers using postal questionnaires can increase response by addressing participants by name on cover letters. The effect appears to be enhanced by including hand-written signatures.

## Competing interests

The author(s) declare that they have no competing interests.

## Authors' contributions

BS and PE designed the study, extracted and analysed the data, interpreted the results and wrote the paper.

## Pre-publication history

The pre-publication history for this paper can be accessed here:



## Supplementary Material

Additional File 1 Table 1 Characteristics of included studies. This table provides summary information about the characteristics of the 14 randomised controlled trials included in the meta analysis. Click here for file
