# Supplementary material for: Personally addressed hand-signed letters increase questionnaire response: a meta-analysis of randomised controlled trials
Source: BMC Health Serv Res. 2006 Sep 5;6:111. doi: 10.1186/1472-6963-6-111 (PMC1574304; doi:10.1186/1472-6963-6-111)
Supplement: Additional File 1 — Table 1 Characteristics of included studies. This table provides summary information about the characteristics of the 14 randomised controlled trials included in the meta analysis. [file 1472-6963-6-111-S1.doc]

**Table 1 Characteristics of included s**tudies

| First author | Year | Country | Number of participants† | Type of participants | Topic | Length | Pre-contact  and follow-up | Anonymity guarantee | Incentives | | Return envelope included | Handwriting |
| --- | --- | --- | --- | --- | --- | --- | --- | --- | --- | --- | --- | --- |
| Childers | 1985 | US | 1500 | Car insurance policy holders | Method of insurance payment | 6 single sides | N/S | Compares anonymous with non-anonymous | N/S | | Yes | N/A |
| Dillman | 1974 | US | 927 | University alumni | Attitudes to university policies | 8 pages, 78 questions | 2 follow-ups, each with the same level of personalisation as the initial mailing | N/S | N/S | | N/S | Signature |
| Gitelson | 1992 | US | 300 | Farm show attendees, non-responders to three mailings | N/S | N/S | 3 prior mailings of questionnaires | N/S | N/S | | N/S | Signature |
| Green | 1989 | US | 600 | Teachers | Application of research findings | 4 single sided pages, 51 questions | 2 follow-ups | N/S | Some offered copy of results, same percentage in each personalis-ation group | | Yes - postage paid | Signature |
| Kerin | 1976 | US | 440 | Corporate presidents | Product recall practices | 4 pages - unclear if single or double sided | N/S | N/S | N/S | | Varied, same percentage in each personalis-ation group | Signature |
| King | 1978 | US | 241 | Banking company CEOs | N/S | 4 pages, unclear if single or double-sided | No | Yes | No | | Yes - reply paid | Signature |
| Martin | 1989 | US | 2000 | University students | Perceived attributes of university | N/S | Varied, same percentage in each personalisation group | N/S | N/S | | Varied, same percentage in each personalisation group | N/A |
| Matteson | 1974 | US | 2123 | Professional organisation members | Significant contributions to literature in field | 2 pages (unclear if double or single sided) | N/S | N/S | N/S | | Yes | Signature |
| Roberts | 1978 | US | 1190 | Dentists | N/S | N/S | 2 mailed follow-ups | N/S | N/S | | N/S | N/A |
| Shin | 1992 | US | 1500 | Faculty managers in universities and colleges | Course evaluation | N/S | No | Yes | No | | N/S | Salutation and signature |
| Weilbacher | 1952 | US | 472 | Professional (Columbia University) | Address and employment information | Postcard questionnaire | No | N/S | No | | N/S | Signature |
| White | 1997 | US | 300 | Marriage and family therapists | Important elements of therapy supervision | 10 single-sided pages, 159 questions | 1 postcard and 1 letter follow-up | N/S | | N/S | Yes | N/A |
| Worthen | 1985 | US | 1000 | School teachers | College course content | N/S | No follow-up for some; Personalisation of follow-up as intervention in others | N/S | | N/S | N/S | Signature |
| Wright | 1984 | New Zealand | 706 | Telephone directory listed people | Garden product use | 7 single-sided pages, 27 questions | 3 mailed follow-ups | No | | Varied - factorial study | Yes -reply paid | N/A |

† Includes undelivered questionnaires and participants allocated to intervention groups not considered in the meta-analysis (Figure 1). N/S = not stated; N/A = not applicable.
